# Editorial: The Microbiological Functionality and Safety of Fermented Foods

**DOI:** 10.3389/fmicb.2022.979329

**Published:** 2022-07-13

**Authors:** Jae-Hyung Mah, Claudia Ruiz-Capillas

**Affiliations:** ^1^Department of Food and Biotechnology, Korea University, Sejong, South Korea; ^2^Institute of Science and Technology of Food and Nutrition (ICTAN), Spanish National Research Council (CSIC), Madrid, Spain

**Keywords:** fermented foods, food functionality, food quality and safety, probiotic microorganisms, spoilage and pathogenic microorganisms, functional compounds, hazardous compounds

A variety of fermented foods have been developed, produced, and consumed worldwide for 100s to 1,000s of years to provide nutrients without being influenced by seasonal availability or the environment (Earnshaw, 1990; Tamang, 2015; Anal, 2019). Due to their health promoting functions, fermented foods are becoming popular all over the world (Tamang, 2015; Sanlier et al., 2019). In the meantime, as humans have been steadily ingesting fermented foods for centuries or millennia and because food scientists have focused mainly on researching the health-promoting effects of the foods, the safety issues of fermented foods have been overlooked (Mah et al., 2019; Behera et al., 2021; Leeuwendaal et al., 2022). Fermented foods are not free from a variety of microbiological safety issues because the foods cannot be processed by conventional sterilization methods. Also, it is difficult to improve the quality of fermented foods because the foods are often produced by traditional methods (Mah et al., 2019; Ashaolu, 2020). For this reason, there is still the risk of food poisoning and spoilage caused by contaminated pathogenic and spoilage microorganisms in fermented foods. Therefore, food scientists must strive to improve the health functionality and safety of fermented foods due to the presence of desirable or undesirable microorganisms (Ashaolu, 2020). This Research Topic focuses on novel information gained from research on the above subjects to provide an interesting overview of *The Microbiological Functionality and Safety of Fermented Foods* and brings together seven original scientific research articles addressing studies on various microorganisms in fermented foods.

Several authors have suggested that the health-promoting functions of fermented foods can be improved by applying probiotic microorganisms as well as optimizing fermentation conditions. As is well-known, of course, to be beneficial human health, probiotics must survive passage through the gastrointestinal tract. Liu et al. assessed the *in vitro* probiotic potential of *Lactobacillus* strains isolated from Chinese artisanal fermented vegetables. Among the isolates with high bile tolerance and low pH tolerance, several strains exhibited great probiotic potential, indicating that they might be excellent candidates for functional food production. Chan et al. studied the effects of prolonged storage, fermentation processes and functional spices on microbial viability of whole fermented foods (tibicos and sauerkraut) from fermentation to digestion. In their study, lactic acid bacteria and yeasts in both foods were able to survive the low pH environment of fermentation, resulting in a sufficient number of microorganisms to be considered probiotics. Ginger and cayenne were found to significantly enhance the survival of lactic acid bacteria during fermentation, storage, and gastrointestinal digestion. Consequently, this study showed that lactic acid bacteria and yeasts in whole fermented foods can be manipulated during production and storage to maximize microbial viability, which in turn may result in higher survival rates when digested and affect the relative abundance of gut bacteria. Jo et al. reported that *Limosilactobacillus fermentum* strains isolated from various plant-based fermented foods had probiotic and health-promoting properties. Of the probiotic strains, *L. fermentum* MG7011 had not only outstanding probiotic properties but also enzymatic activities of amylase and phytase. Based on the results, they suggested that this strain can be used as a suitable probiotic starter for the fermentation of a rice beverage. Besides, some probiotic bacteria may be used to prevent infection by pathogens such as viruses. For instance, S-layer proteins (SLPs) derived from lactic acid bacterial cell walls have been considered as antiviral compounds. Kawahara et al. found that SLPs of *Lactobacillus crispatus* strain KT-11 had an inhibitory effect on rotavirus infection. Since the SLPs had resistance to digestion with gastric juice, the authors suggested that the antiviral compounds may prevent rotavirus infection in the gastrointestinal environment.

Meanwhile, it has been known that fermented foods may have safety issues due to microbial metabolites such as ethyl carbamate, biogenic amines, and microbial toxins (Verbeke et al., 2015; Ruiz-Capillas and Herrero, 2019). Therefore, the microorganisms causing such problems must be identified or determined, monitored, and controlled. Berthoud et al. reported the ability of *Paucilactobacillus wasatchensis* WDC04 to produce cadaverine and putrescine in culture medium supplemented with lysine and ornithine, as well as in a model cheese produced with adjunct culture of *P. wasatchensis*. Besides, they identified the putative amino acid decarboxylase genes and evaluated the decarboxylation activity of recombinant proteins, as well as their substrate preference. Thus, it turned out that the lysine decarboxylase genes of lactic acid bacteria could explain high levels of cadaverine in cheese. Zhou et al. explored the main environmental factors (salinity, temperature, anaerobic conditions, etc.) for citrulline formation by the two key strains of *Pediococcus acidilactici* and *Weissella confusa* isolated from soy sauce moromi and proposed a targeted way to control citrulline formation in soy sauce, with the aim to explore its application potential in ethyl carbamate control in soy sauce. Consequently, this study proposed a potential method to control citrulline formation by lowering fermentation temperature or by adding polyphenols. Apart from harmful metabolite-producing lactic acid bacteria, pathogenic bacteria including spore-forming bacteria also cause important safety issues in fermented foods. To reduce the risk, a sufficient understanding of the physicochemical and structural properties of pathogenic bacteria is required to establish a strategy. As part of the efforts, Sinnelä et al. reported that the heat resistance of the spores of *Bacillus cereus, B. licheniformis, B. coagulans*, and *B. subtilis*, varied depending on the species. Based on the research, it turned out that the spores of *B. cereus* had a significantly lower heat resistance than those of the other *Bacillus* species. The authors also suggested that the supplementation of specific concentrations of calcium and manganese could enhance the heat resistance of beneficial *Bacillus* spores while lowering that of undesirable *Bacillus* spores (particularly *B. cereus*).

In summary, this Research Topic introduces original research articles describing the newest knowledge and the latest strategies for the improvement of the health functionality and safety of fermented foods. As proposed in most articles, more attention may need to be paid to the desirable and undesirable roles of various microorganisms in fermented foods.

## Author contributions

J-HM: conceptualization, original draft preparation, review, and editing. CR-C: original draft preparation and review. Both authors listed have made a substantial, direct, and intellectual contribution to the work and approved it for publication.

## Funding

J-HM was supported by a grant from Korea University. CR-C was supported by the Spanish Ministry of Science and Innovation (PID2019-107542RB-C21).

## Conflict of interest

The authors declare that the research was conducted in the absence of any commercial or financial relationships that could be construed as a potential conflict of interest.

## Publisher's note

All claims expressed in this article are solely those of the authors and do not necessarily represent those of their affiliated organizations, or those of the publisher, the editors and the reviewers. Any product that may be evaluated in this article, or claim that may be made by its manufacturer, is not guaranteed or endorsed by the publisher.
